# Endometrial Cancer: A Pilot Study of the Tissue Microbiota

**DOI:** 10.3390/microorganisms12061090

**Published:** 2024-05-28

**Authors:** Claudia Leoni, Lorenzo Vinci, Marinella Marzano, Anna Maria D’Erchia, Miriam Dellino, Sharon Natasha Cox, Amerigo Vitagliano, Grazia Visci, Elisabetta Notario, Ermes Filomena, Ettore Cicinelli, Graziano Pesole, Luigi Ruggiero Ceci

**Affiliations:** 1 Institute of Biomembranes, Bioenergetics and Molecular Biotechnologies, National Research Council (CNR), Via Amendola n. 122/O, 70126 Bari, Italy; m.marzano@ibiom.cnr.it (M.M.); e.notario@ibiom.cnr.it (E.N.); 2 2nd Unit of Obstetrics and Gynaecology, Department of Biomedical Science and Human Oncology, University of Bari “A. Moro”, Piazza G. Cesare, 70124 Bari, Italy; dottvincilorenzo@gmail.com (L.V.); miriamdellino@hotmail.com (M.D.); amerigo.vitagliano@uniba.it (A.V.); ettore.cicinelli@uniba.it (E.C.); 3 Department of Biosciences, Biotechnologies and Environment, University of Bari A. Moro, Via Orabona n. 4, 70126 Bari, Italy; annamaria.derchia@uniba.it (A.M.D.); sharonnatasha.cox@uniba.it (S.N.C.); grazia.visci@uniba.it (G.V.); ermes.filomena@gmail.com (E.F.); g.pesole@ibiom.cnr.it (G.P.)

**Keywords:** endometrial cancer, microbiota dysbiosis, ddPCR, 16S rRNA gene, metabarcoding

## Abstract

Background: The endometrium remains a difficult tissue for the analysis of microbiota, mainly due to the low bacterial presence and the sampling procedures. Among its pathologies, endometrial cancer has not yet been completely investigated for its relationship with microbiota composition. In this work, we report on possible correlations between endometrial microbiota dysbiosis and endometrial cancer. Methods: Women with endometrial cancer at various stages of tumor progression were enrolled together with women with a benign polymyomatous uterus as the control. Analyses were performed using biopsies collected at two specific endometrial sites during the surgery. This study adopted two approaches: the absolute quantification of the bacterial load, using droplet digital PCR (ddPCR), and the analysis of the bacterial composition, using a deep metabarcoding NGS procedure. Results: ddPCR provided the first-ever assessment of the absolute quantification of bacterial DNA in the endometrium, confirming a generally low microbial abundance. Metabarcoding analysis revealed a different microbiota distribution in the two endometrial sites, regardless of pathology, accompanied by an overall higher prevalence of pathogenic bacterial genera in cancerous tissues. Conclusions: These results pave the way for future studies aimed at identifying potential biomarkers and gaining a deeper understanding of the role of bacteria associated with tumors.

## 1. Introduction

The research carried out during the Human Microbiome Project (HMP) emphasized the significance of microorganisms and their genomes in various human niches (the mouth, skin, lungs, gut, and genitourinary area) and underlined their close correlation with human health and disease [1]. The human microbiota is made up of different microbial taxa that coexist in the human body and amount to 100 trillion microbes [2]. Accordingly, the microbial population in the female reproductive system has long been investigated for its role in determining health and disease states. Lactic acid-producing microbes play an important role in determining the microbial community of the vaginal microbiome and have been shown to protect against infections [3]. Alterations in the microbiota are involved in the development of up to 15% of tumors [4], and an imbalance or dysbiosis of microbial populations in the female reproductive tract may favor endometrial cancer, the 15th most common cancer worldwide [5]. The uterus is characterized by a low-abundance microbiome, containing between 100- and 10.000-times fewer microorganisms than its vaginal counterpart [6,7,8,9], which presents an obstacle for its comprehensive characterization [10]. In addition, studying the uterine microbiome is challenging due to potential contaminations from adjacent compartments during sampling procedures such as transvaginal sampling [11]. Direct sampling of the microbiota from the uterus is only possible in patients undergoing surgical opening and uterus removal, typically in cases with endometrial cancer [9,12,13,14]. While two of the related studies identified variations in bacterial composition, with an increased presence of species like *Porphyromonas somerae* and *Atopobium vaginae* in endometrial cancer patients [13,14], more recently, Gressel et al. found a depletion in *Lactobacillus* and an increase in *Pseudomonas* in patients with serous endometrial cancers compared with the control [12]. These inconsistent findings are not surprising when considering variables such as the different bacterial taxa levels examined (e.g., phylum, genus, and species), the analysis of different regions of the 16S rRNA gene (e.g., V3–V5 and V4), variations in patients’ statuses, and, finally, the absence of specifications regarding the endometrial sampling region. Consequently, the scenario of the human uterine microbiota and its dynamics under physiological or pathological conditions are still unclear. To fill this gap, in this work, we aimed to explore the potential correlation between microbiota dysbiosis and the development of endometrial cancer. Specifically, we investigated the endometrial microbiota profiles in women with endometrial cancer pathology across different levels of tumor differentiation and in women with a polymyomatous uterus, serving as control cases [15,16]. Furthermore, to evaluate whether the endometrial microbiota could have different compositions due to influences from non-uterine sources (e.g., vaginal transmission and blood flow), we analyzed the microbiota of two distinct sites of the endometrium.

Firstly, we employed droplet digital PCR (ddPCR) analysis on the two selected endometrial sites to provide a more detailed representation of the endometrial microbiota. This approach enabled the assessment of the absolute amount of bacterial presence, confirming, for the first time, the low abundance of microbial presence in endometrial tissues. Subsequently, the microbiota of the endometrial samples was investigated by the NGS amplicon-based application, targeting the V5–V6 hypervariable regions of the 16S rRNA gene in both cancer and control cases and in two distinct uterine sites. A higher number of genera were identified in cancer cases, potentially serving as targets for further analysis to establish relationships with cancer pathology.

## 2. Materials and Methods

### 2.1. Study Cohort and Sample Collection

Women being treated at the 2nd Unit of Obstetrics and Gynecology, the University of Bari, the Polyclinic University-Hospital, Bari (Italy), for suspected endometrial cancer, were considered for this study. Histological analysis allowed for a clear distinction between women affected by endometrial cancer with different differentiation levels and women affected by a polymyomatous uterus. Eight cases of endometrial cancer constituted the study group, while six cases of polymyomatous uterus were enrolled as the control group. This study was approved by the Institutional Ethical Committee of the Polyclinic University Hospital, Bari (Italy), and informed consent was obtained from each patient (prot. no. 0049155—24 May 2023). There were no conflicts of interest associated with this study. The sampling of endometrial biopsies was carried out between January and June 2022. Biopsies were obtained under sterile conditions in the operating room at the time of hysterectomy using disposable scalpels and sterile forceps. The incision was made at the level of the coronal uterine opening and excluding the cervical canal, thus distinguishing the anterior and posterior uterine wall. In the control group, the anterior wall of the uterus was always considered to standardize sampling, while in the group of carcinomas, the uterine wall with the greatest presence of tumor material was considered. The sampling was performed at two different points at the level of the endometrium, positioned at 12 o’clock (site 12) and 3 o’clock respectively (site 3) (Figure S1 in the Supplementary Materials). Biopsies of about 7 mm long and 2 mm wide were obtained from the base of the neoplastic tissues. The tissue samples were stored at −80 °C until further analyses. Demographic and clinical data were expressed as means ± the standard error of the mean (SEM), and a two-tailed Student’s *t*-test was used to assess differences between the two groups. Qualitative variables were summarized as counts and percentages, and comparisons between independent groups were performed by Fisher’s exact test.

### 2.2. DNA Isolation

DNA from endometrial samples was extracted using the FastDNA Spin Kit for soil (MP Biomedicals, Irvine, CA, USA), according to the manufacturer’s instructions, and eluted in 100 µL of sterile distilled water. A negative control for the extraction was carried out using the same reagents and tubes supplied with the kit and without tissue. The eluted DNA was qualitatively analyzed by electrophoretic analysis and quantitatively by spectrophotometric and fluorometric assays using a NanoDrop ND-1000 spectrophotometer and the Qubit dsDNA HS assay kit (Thermo Fisher Scientific, Waltham, MA, USA), as described in consolidated procedures, respectively [11].

### 2.3. Droplet Digital PCR Assay (ddPCR) and Estimation of Bacterial DNA Content

The total number of 16S rRNA copies present in the eDNA was determined by ddPCR (Bio-Rad, Hercules, CA, USA), using universal primers targeting the V5–V6 regions of the gene. The reaction was set up with a final volume of 22 µL, combining 50 ng of eDNA with 11 µL of 2X Evagreen Supermix and primers at a 100 mM final concentration. Each eDNA sample was amplified in triplicate, and for each experiment, a negative control (no template control) was used. DNA amplification droplets were produced by emulsion with a QX200 droplet generator (Bio-Rad Hercules, CA, USA) according to the manufacturer’s instructions. PCR reactions were carried out using a BioRad T100 thermal cycler, according to already reported amplification profiles [17]. QuantaSoft version 7.4.1 software (Bio-Rad, Hercules, CA, USA) was used for absolute quantification, and the negative/positive thresholds were set manually. The output results were expressed in 16S rRNA gene copies/ng. For the estimation of the bacterial DNA present in 1 ng of DNA, we considered the following values: the average copy number of the 16S rRNA gene per bacterial genome was 4 [18]; the average size of the bacterial genome was 5 Mbp [19]; and the average mass of 1 base pair (obtained starting from the average molar mass of 1 bp equal to 650 g/mole) was 1.1 × 10^−12^ ng. Accordingly, the following equation was applied:

Bacterial DNA/ng DNA = (5 × 10^6^ (bp/bacterial genome) 1.1 × 10^−12^ (ng/bp)/4 (16S rRNA copy number/bacterial genome) = 1.4 × 10^−6^ ng bacterial DNA/ng DNA (1)

Pearson’s correlation analysis was used to measure the correlation between the absolute quantification of the 16S rRNA gene presence at endometrium sites 3 and 12. A two-tailed Student’s t-test was used to assess the differences between the cancer and control cases in the two different regions. A *p*-value of <0.05 was considered statistically significant. All statistical analyses and graphs were generated with GraphPad Prism 8.0 (GraphPad Software, San Diego, CA, USA).

### 2.4. Metabarcoding Amplicon Library Preparation and Illumina-Based Sequencing

Starting with each eDNA sample, the bacterial amplicon libraries were prepared by amplifying the V5–V6 hypervariable regions of the 16S rRNA gene using the primers B-V5 and A-V6 [11,20]. An amount of 100 ng of each DNA sample was used for a two-step amplification reaction, as already reported for the analysis of other microbiota [11]. RNase/DNase-free molecular biology-grade water (Ambion) was used as a negative control for the PCR amplifications. The obtained amplicons were purified and pooled in an equimolar ratio and subjected to 2 × 250 bp paired-end (PE) sequencing on the Illumina MiSeq platform (Illumina). The phage PhiX genomic DNA library was added to the mix and co-sequenced as the internal control for the Illumina sequencing run and to increase the genetic diversity.

### 2.5. Bioinformatics Analysis

The raw sequences were analyzed using the QIIME2 pipeline (version 2022.2; https://qiime2.org, accessed on 10 October 2023) [21], relying on the ASVs’ (amplicon sequence variants) inference and their taxonomic classification. The PE sequences were imported, and the Illumina adapters were removed using cutadapt. Following the evaluation of sequence quality, the PE sequences were denoised by the DADA2 plugin [22]. In this step, the specific primer pairs were trimmed, and the raw reads were filtered according to the observed expected error. Then, the obtained ASVs were taxonomically annotated by using the feature classifier (method: classify_sklearn) plugin [23] against the Silva database (release 138) [24]. The phylogenetic inference was achieved by using the align-to-tree-mafft-fasttree plugin: a multiple sequence alignment of the ASVs’ sequences was obtained by using MAFFT [25], and the phylogenetic tree was inferred by the maximum-likelihood procedure implemented in Fasttree 2 [26]. The R packages phyloseq [27] (https://joey711.github.io/phyloseq/, accessed on 10 October 2023) and vegan [28] were used to measure the alpha- and beta-diversity [29]. The ASVs’ counts were normalized to 115,000 sequences per sample by using rarefaction, and the alpha diversity inference (i.e., intra-sample diversity) was measured with the Shannon and inverse Simpson indexes. Statistical differences in the alpha-diversity indexes were measured using the Wilcoxon test (*p* < 0.05 was considered statistically significant). The beta-diversity (i.e., inter-sample diversity) was measured with the Aitchison distance by transforming the data through the CLR (centered log-ratio) [28], and then PCoA (principal coordinates analysis) was applied to simplify the data interpretation. PERMANOVA analysis was used to infer the explained variability in the β-diversity data by applying 999 permutations. Statistically significant traits in the microbial composition of each experimental group (control and cancer) were evaluated at different taxonomic ranks using the LDA effect size (LEfSe) method [30].

## 3. Results

### 3.1. Patients, Metadata, and Sampling Sites

Eight patients with a diagnosis of endometrial cancer and six patients with a diagnosis of a polymyomatous uterus (representing the control cases) were subjected to DNA analysis of their endometrial microbiota. Polymyomatous uterus is the most common benign condition requiring hysterectomy, allowing the same sampling conditions that occur for the collection of tumor biopsies.

The patients enrolled in this study had a wide epidemiological variability. Besides tumor differentiation, the main variables were age, BMI, pregnancies, miscarriages, smoking, and menopausal status (Table 1). As the control, patients who underwent hysterectomies for a polymyomatous uterus were considered. In carcinoma cases, all patients underwent hysterectomies with a preoperative diagnosis of endometrial carcinoma. Patients who underwent surgery with an intracervical manipulator during the procedure, who received antibiotic therapy in the last 60 days before surgery, or who had other ongoing oncological or infective diseases were excluded. The carcinoma tissues subjected to histological analysis showed different levels of tumor differentiation: G1, corresponding to well-differentiated cells; G2, corresponding to moderately differentiated cells; and G3, corresponding to poorly differentiated cells.

Biopsies were taken at two specific sites of the endometrium, positioned at 12 o’clock (site 12) and 3 o’clock (site 3), respectively (Figure S1 in Supplementary Materials).

### 3.2. Absolute Quantification of Bacterial DNA

Droplet digital PCR (ddPCR) analysis was carried out to quantify the copy number of the 16S rRNA gene present in the endometrial DNA (or “environmental DNA” (eDNA)) isolated from the two sites (3 and 12) of both cancer and control cases. Amplifications were carried out using the universal primer pair targeting the V5–V6 hypervariable regions of the gene. The data obtained (Table 2) indicated a relatively low abundance of the 16S rRNA gene for both the control and cancer cases (between 0.5 and 2.8 copies/ng of eDNA). These findings provide an approximate indication of the bacterial content in the endometrial samples. Considering the average values reported in Table 2 of the 16S rRNA gene copies per 1 ng of eDNA and the average value of 1.4 × 10^−6^ ng of bacterial DNA/ng DNA (see Equation (1) in the Materials and Methods), the amount of bacterial DNA in 1 ng of total eDNA ranged between 0.7 and 3.9 fg (femtograms) (Table 2).

A significant correlation was found between the absolute quantification of the 16S rRNA gene in K3 and K12 cancer cases (Pearson’s r = 0.8241; *p* = 0.0059), while no correlation was found between C3 and C12 control cases (Pearson’s r = 0.2293; *p* = 0.33; see Figure S2 in the Supplementary Materials). The same results were obtained considering normalized bacterial DNA (Figure S3 in the Supplementary Materials). We performed unpaired *t*-tests to assess if there were statistically significant differences between cancer and control cases in the two different regions, and we found no statistically significant differences.

### 3.3. Metabarcoding Analysis

**Figure 1:**
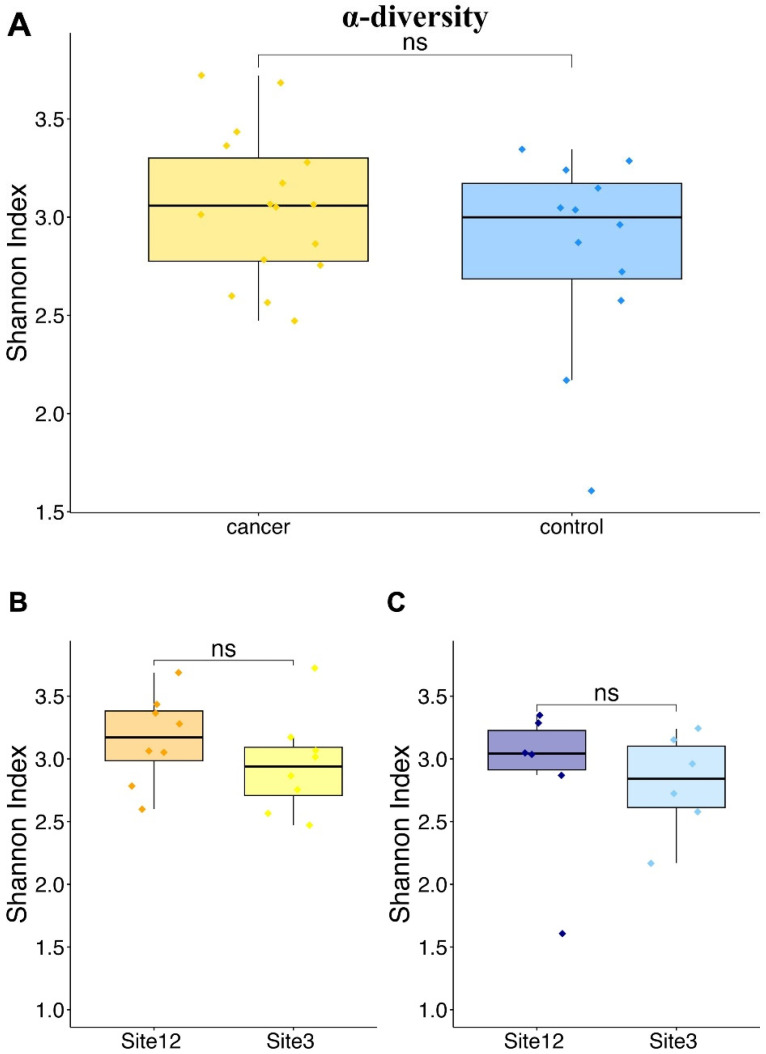
Shannon index α-diversity values. (**A**) Shannon index values for control and cancer cases; (**B**) Shannon index values for sites 12 and 3 in cancer cases; and (**C**) Shannon index values for sites 12 and 3 in control cases (ns, not statistically significant).

To analyze the bacterial population in each sample, we performed the amplification and sequencing of the V5–V6 hypervariable regions of the 16S rRNA gene according to a previously established procedure [31]. After trimming raw sequences and removing chimera and Phix sequences, we obtained approximately 6.3 million total reads, averaging at 210 thousand reads per sample. All sequencing raw data (corresponding to 14 patients for the two sampling sites 3 and 12) were deposited in the SRA (Short Read Archive) repository with accession number PRJNA1017407. The α-diversity was measured by using the Shannon index (Figure 1) and the inverse Simpson index (Figure S4 in the Supplementary Materials) starting from normalized ASV counts. The analysis showed similar levels of biodiversity with no statistically significant differences between the two groups (Figure 1A). The same result was obtained when comparing site 3 and site 12 for the control and cancer cases (Figure 1B,C).

**Figure 2:**
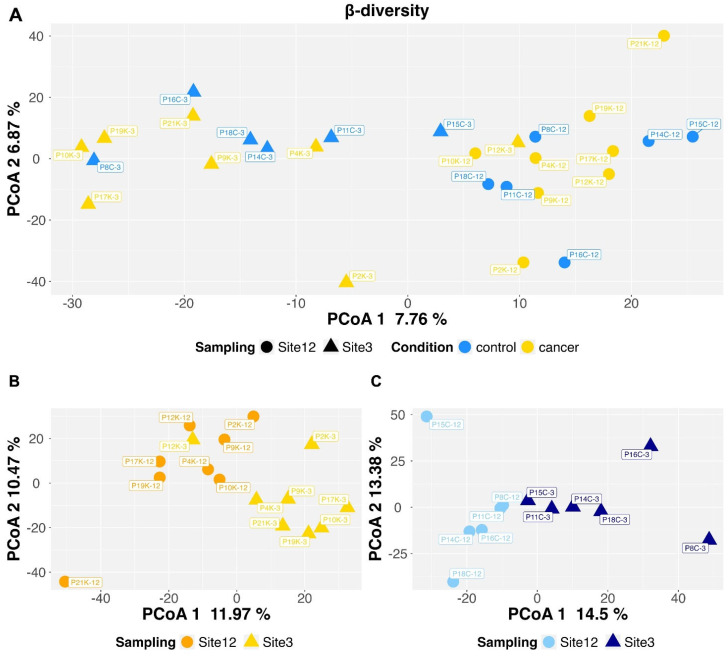
Principal coordinates analysis (PCoA) representation by applying the Aitchison distance to CLR-transformed data. Panel (**A**): overall data representation. Panels (**B**,**C**): distinguished PCoA analysis for cancer (PERMANOVA *p*-value = 0.0005) and control (PERMANOVA *p*-value = 0.0013) cases, respectively.

To evaluate the inter-sample diversity between the different conditions (control vs. cancer; site 3 vs. site 12), β-diversity analysis was performed. Considering both components, no separation was observed between the control and cancer group samples (7.76% for PCoA1; 6.87% for PCoA2) (Figure 2A). However, based on the first component, within the same group (control or cancer), the samples clustered separately according to the endometrial region (sites 3 and 12) (Figure 2B,C).

### 3.4. Taxonomic Composition of the Endometrial Samples

**Figure 3:**
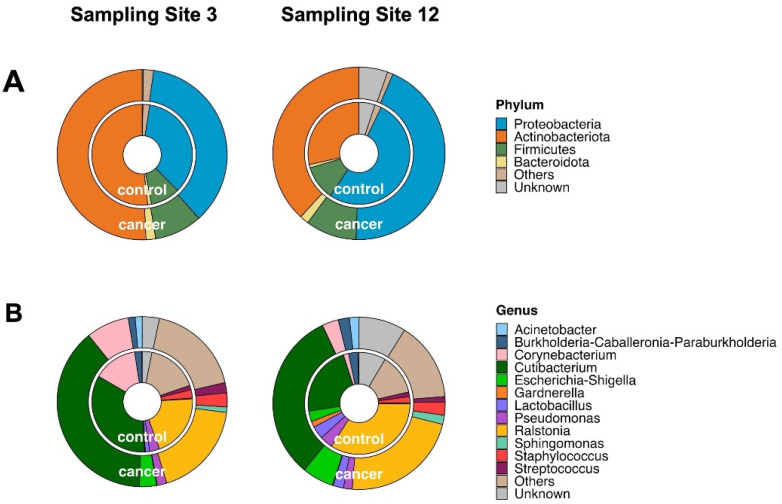
Donut charts representing the taxonomic assignment of the microbiome at the phylum (**A**) and genus ranks (**B**) for sampling sites 3 and 12. Values represent the averages across all the samples. Groups with relative abundances of <1.0% were joined as “Others”.

The bacterial taxonomic assignments and relative abundances for the control and cancer cases at the phylum and genus levels in the two endometrial regions (sites 3 and 12) are shown as donut charts (Figure 3). Only taxa with a relative abundance (RA) equal to or higher than 1% were plotted; otherwise, they were collapsed into “Others”.

At the phylum level (Figure 3A), *Proteobacteria* and *Actinobacteria* were the most abundant phyla in both the control and cancer groups, followed by *Firmicutes* and *Bacteriota*. Considering the sampling sites, *Proteobacteria* were more abundant at site 12 (50% in control cases and 42% in cancer cases) with respect to site 3 (37% in control cases and 36% in cancer cases). Meanwhile, *Actinobacteria* were more present at site 3 (49% in control cases and 50% in cancer cases) than at site 12 (27% in control cases and 36% in cancer cases). The phylum *Firmicutes* was less abundant, present at the same percentage (9%) at sites 3 and 12 in both conditions.

At the genus level (Figure 3B), except for *Gardenella*, the same bacterial genera were found in cancer and control cases. The genus *Gardnerella* was present only in one control case (sample 14C), mostly at site 12 rather than at site 3 (10% and 1%, respectively) (see Table S1 in the Supplementary Materials).

The common genus present in cancer and control cases was *Cutibacterium*, with the highest RAs in cancer cases (38% at site 3 and 31% at site 12) compared with control cases (34% at site 3 and 21% at site 12); inversely, *Ralstonia* was present at higher RAs in control cases (20% at site 3 and 33% at site 12) compared with cancer cases (18% at site 3 and 21% at site 12). *Corynebacterium* showed an inconsistent distribution: in cancer cases, it presented a lower RA than in controls at site 3 (8% vs. 14%), while its RA was higher than in controls at site 12 (3% and 2%, respectively) (Table S1 in the Supplementary Materials).

The genera Acinetobacter, Burkholderia-Caballeronia-Paraburkholderia, Escherichia-Shigella, Lactobacillus, Pseudomonas, Sphingomonas, Staphylococcus, and Streptococcus were not present in all the samples and had different RAs at the two sampling sites in both cancer and control cases (Table S1 in the Supplementary Materials).

The LefSe statistical study, conducted at the genus level for all samples, identified some taxa associated with cancer and control conditions, even if with abundances of lower than 1% (Table S2 in the Supplementary Materials).

### 3.5. Main Microbial Differences between Samples

To facilitate the comparison of the microbiota compositions in the cancer and control cases at sites 3 and 12, a summary table was prepared containing only the bacterial genera identified in more than 50% of the examined samples (Table 3). The average RAs were calculated by considering all the values obtained in the metabarcoding analysis. Each distribution value represents the percentage of subjects in which the genus RA was ≥1.0% and was considered only if it was ≥50%.

## 4. Discussion

This study described the composition of the human uterine microbiota of 14 patients, including 8 patients with endometrial cancer and 6 patients with a polymyomatous uterus. Sampling was conducted at two distinct sites of the endometrium, indicated as sites 3 and 12, to investigate the potential variations in the microbiota composition deriving from adjacent organs and blood flow. Vaginal contaminations of the endometrial microbiota have already been reported [10], but their effect on different regions of the endometrium has never been investigated. We also carried out the absolute quantification of bacterial abundance at the two sites. For this purpose, ddPCR assays were performed to detect and quantify the bacterial 16S rRNA gene. ddPCR is widely used for the absolute quantification of DNA with high sensitivity in different types of biological samples [17]. Accordingly, it can be used for the quantification of bacterial DNA in heterogeneous samples. The technique requires a careful selection of primers targeting the 16S rRNA gene to effectively sample prokaryotic diversity and ensure accurate abundance estimates while minimizing bias. The experiments showed the presence of 0.5–2.8 copies of the 16S rRNA gene per 1 ng of eDNA in both cancer and control cases (Table 2). These findings provide an approximate indication of the bacterial content in the endometrial samples, estimated to range between 0.7 and 3.9 fg/ng eDNA. This marks the first report of the absolute quantification of bacterial genomic DNA quantification in endometrial samples. Based on the ddPCR results, the presence of a cancerous condition does not seem to have an overall impact on the bacterial presence. However, in cancerous tissues, a positive correlation with the absolute bacterial DNA abundance at the two sampled sites (3 and 12) was found, whereas no correlation was found in control tissues. This suggests that there may be a diffuse microbial dysbiosis associated with the cancerous state, potentially induced by specific bacterial populations in the tumor microenvironment or in response to cancer-associated changes. Therefore, we investigated the composition of the bacterial microbiota and found some differences between the two conditions studied. These differences were evident through metabarcoding NGS analysis targeting the V5–V6 hypervariable region of the 16S rRNA gene. In control cases, 12 genera were identified with an RA of >1%, whereas in cancer cases, 11 genera were identified (Table S1 in the Supplementary Materials). Although the observed genera exhibited different abundances, elucidating the specific variations between the samples is not straightforward. In pilot studies such as the one presented here, the interpretation of observed data may be strongly influenced by the values of even a single patient. Therefore, to have a more general description of the microbiota distribution, we selected the bacterial genera that were present in more than 50% of the examined samples (Table 3). Among the genera identified, three (*Corynebacterium*, *Ralstonia*, and *Cutibacterium*) were present in all cases (100%), while others had lower distribution values (Table 3). Notably, several genera with higher distribution values (of at least 50%), including *Corynebacterium, Cutibacterium*, *Staphylococcus*, *Streptococcus,* and *Anaerococcus* are typical of the human microbiota [10,32,33,34,35] and can be considered, at least under the particular conditions of this study, as part of the human core endometrial microbiota. Notably, these five genera (*Cutibacterium*, *Escherichia-Shigella*, *Staphylococcus*, *Streptococcus*, and *Corynebacterium*) were also detected in more than 50% of samples in a previous study of the endometrial microbiota in women undergoing elective cesarean delivery [31]. However, the presence of the genera *Burkholderia* and *Ralstonia* likely indicated infections or contaminations, common in hospital samples [31].

Some other interesting observations can be drawn from the data presented in Table 3. Nine genera (*Sphingomonas*, *Acinetobacter*, *Actinomyces*, *Escherichia-Shigella*, *Hydrogenophaga*, *Methylobacterium-Methylorubrum*, *Neisseria*, *Rhodococcus*, and *Rothia*) were specific to the endometrial microbiota in cancerous tissues, with no genera exclusively present in control tissues. Among these, the *Sphingomonas* [36], *Acinetobacter* [37], *Actinomyces* [38], *Escherichia-Shigella* [39], *Neisseria* [40], and *Rothia* [41] genera have been associated with various human pathologies, while *Hydrogenophaga* [42] and *Rhodococcus* [43] have been identified in hospital environments. According to this analysis, the presence of these nine genera makes the endometrial microbiota of cancer patients richer in bacterial diversity compared with non-cancerous tissues. However, there are currently no indications of any functional relationships between these bacteria and the cancer pathology, even if some genera have been identified in other cancers: the genus *Escherichia-Shigella* is considered to be a potential pathogen associated with colorectal cancer [44] and the inflammatory status of the intestinal tract [45]; the genus *Hydrogenophaga* was found to be enriched in the breast tissues of women with invasive breast cancer [46]; and the genus *Methylobacterium-Methylorubrum* was detected as being significantly increased in gastric cancer tissues [47]. The species *Actinomyces radius* has recently been identified as a possible marker of oral cancers [48]. Further analysis of these specific bacterial genera in at-risk patients would be of interest to confirm any potential relationship with the pathology.

Furthermore, the specific presence of most of these genera in only one of the two sampled endometrial regions is also interesting. It indicates a non-homogeneous distribution of the bacteria, possibly influenced by different tissue compositions or bacterial translocation from other organs (e.g., the vagina). This finding indicates the need to precisely define the endometrial sampling site in future microbiota studies. Moreover, this aspect may help elucidate the differences observed between our study and previous similar studies on microbiome composition in endometrial cancer [12,13,14].

This pilot study gives a snapshot of the microbiota composition in endometrial cancer. However, a complete analysis of its functional relationship with cancer development is far from being reached. Indeed, the activity and role of the microbial population can only be understood if investigated more thoroughly at the functional level and in conjunction with the host response. This would require a complete system analysis based on multiomics studies of both microbial and host counterparts [49,50].

## 5. Conclusions

In this study, the endometrial microbiota of women affected by endometrial cancer, or benign uterine fibroids, was characterized. For the first time, the relative abundance of the bacterial DNA in endometrial biopsies was evaluated by ddPCR, which confirmed the very low abundance of bacteria in this organ. Nevertheless, a deep metabarcoding NGS approach allowed us to characterize the microbiota profiles in two distinct endometrial sites (referred to as sites 3 and 12, respectively) in both cancer and control cases. This analysis revealed differences in the microbiota distributions between the two sites, regardless of the pathology. Moreover, cancerous tissues showed a higher presence of pathogen bacterial genera. These results suggest the potential association of tumor biomarkers with bacterial dysbiosis profiles. In addition, future functional studies on bacteria associated with cancerous tissues will help clarify their role in disease progression.

## Figures and Tables

**Table 1:** Clinical and demographic features of 8 cancer patients and 6 controls included in this study. Data are expressed as means ± standard error mean (SEM); n., number of cases; n.a., not applicable; ^#^ two-tailed Student’s *t*-test; ^§^ Fisher’s exact Test.

	Cancer	Control	*p*-Value
n.	8	6	
Age (years), mean (SEM)	65.13 (1.17)	43.06 (0.98)	0.0011 ^#^
Weight (kg), mean (SEM)	78.38 (3.08)	69.21 (2.11)	0.8338 ^#^
Height (cm), mean (SEM)	158.63 (0.54)	129.15 (1.06)	0.3600 ^#^
BMI, mean (SEM)	31.15 (1.19)	26.88 (0.93)	0.6902 ^#^
Pregnancies, mean (SEM)	3.875 (0.50)	2.94 (0.14)	0.4295 ^#^
Miscarriages, mean (SEM)	2.13 (0.35)	1.33 (0.07)	0.4262 ^#^
Smoking, n. (%)	1 (12.5)	0 (0)	0.9999 ^§^
Menopause, n. (%)	7 (87.5)	1 (16.7)	0.0256 ^§^
Tumor grade			
G1, n. (%)	2 (25)	n.a.	
G2, n. (%)	4 (50)	n.a.	
G3, n. (%)	2 (25)	n.a.	
FIGO grade			
1A, n. (%)	5 (63)	n.a.	
1B, n. (%)	3 (38)	n.a.	
pT			
pTIa, n. (%)	7 (88)	n.a.	
pTIb, n. (%)	1 (13)	n.a.	
Histological type			
Endometrioid, n. (%)	7 (88)	n.a.	
Squamous, n. (%)	1 (13)	n.a.	

**Table 2:** ddPCR-measured total 16S rRNA gene copy number in 1 ng of total eDNA for control cases (C) and cancer cases (K) and for distinct endometrial sampling sites, indicated as site_3 and site_12. Values are the averages from triplicate experiments. S.D. is standard deviation. Negative controls gave a zero copy number for the 16S rRNA gene.

	Copies of 16S rRNA Gene/ng of eDNA				Bacterial DNA fg/ng of eDNA	
Patients	Site_3	S.D.	Site_12	S.D.	Site 3	Site 12
P8_C	2.8	2.5	1.3	0.6	3.9	1.8
P11_C	1.0	0.9	0.5	0.4	1.4	0.7
P14_C	0.5	0.2	1.6	1.2	0.7	2.2
P15_C	2.1	0.7	2.0	0.8	2.9	2.8
P16_C	1.4	0.3	0.5	0.6	2.0	0.7
P18_C	1.0	0.5	1.3	1.6	1.4	1.8
P2_K	2.1	0.6	1.4	0.7	2.9	2.0
P4_K	1.3	2.3	1.0	0.3	1.8	1.4
P9_K	1.3	1.4	0.8	0.1	1.8	1.1
P10_K	1.1	1.3	0.8	0.7	1.5	1.1
P12_K	2.1	0.6	2.5	0.5	2.9	3.5
P17_K	1.2	1.9	0.6	0.5	1.7	0.8
P19_K	0.7	0.6	0.7	0.1	1.0	1.0
P21_K	1.8	1.2	2.1	1.0	2.5	2.9

**Table 3:** Bacterial genera identified in endometrial microbiota. Average distribution of bacterial genera across cancer and control cases, sampled at sites 3 and 12, expressed as a percentage of the total population. Bacterial genera are listed along with their average distribution percentage.

	Cancer Site 3		Cancer Site 12		Control Site 3		Control Site 12	
	Average	Distribution	Average	Distribution	Average	Distribution	Average	Distribution
*Corynebacterium*	8%	100%	3%	100%	14%	100%	2%	100%
*Ralstonia*	18%	100%	21%	100%	20%	100%	33%	100%
*Pseudomonas*			2%	88%	5%	67%	6%	67%
*Cutibacterium*	38%	100%	31%	100%	34%	100%	21%	100%
*Staphylococcus*	3%	100%	3%	88%	3%	100%	2%	100%
*Streptococcus*	2%	100%	1%	63%	2%	67%	2%	67%
*Anaerococcus*	1%	63%	1%	50%	1%	50%	1%	50%
*Burkholderia-Caballeronia-Paraburkholderia*	1%	88%	2%	100%	3%	83%	3%	100%
*Sphingomonas*	1%	63%	2%	75%				
*Acinetobacter*			3%	50%				
*Actinomyces*	1%	50%						
*Escherichia-Shigella*			6%	88%				
*Hydrogenophaga*	1%	50%						
*Lactobacillus*			2%	88%			4%	100%
*Methylobacterium-Methylorubrum*	1%	50%	1%	63%				
*Neisseria*	1%	50%						
*Rhodococcus*			1%	50%				
*Rothia*	1%	50%						
**Total**	**78%**		**79%**		**81%**		**72%**	
**Different genera**	**13**		**14**		**8**		**9**	

## Data Availability

The original contributions presented in this study are included in this article/the Supplementary Materials and have been submitted to a public database (SRA PRJNA1017407). Further inquiries can be directed to the corresponding author.

## Supplementary Materials

The following supporting information can be downloaded at https://www.mdpi.com/article/10.3390/microorganisms12061090/s1. Figure S1: Endometrial sampling points; Figure S2: Correlation analysis for absolute quantification of the 16S rRNA gene’s presence at two endometrial sites; Figure S3: Correlation analysis for absolute quantification of bacterial DNA in 1 ng of total eDNA at two endometrial sites; Figure S4: Shannon–Simpson index α-diversity values for control and cancer cases; Table S1: Bacterial genera with a percentage of presence of ≥1% for all samples; Table S2: LefSe analysis at genus level for control and cancer cases.
