# Supplementary material for: Endometrial Cancer: A Pilot Study of the Tissue Microbiota
Source: Microorganisms. 2024 May 28;12(6):1090. doi: 10.3390/microorganisms12061090 (PMC11205883; doi:10.3390/microorganisms12061090)
Supplement: Supplementary file 1 [file microorganisms-12-01090-s001.zip › microorganisms-3008244-supplementary.pdf]

Supplementary material.

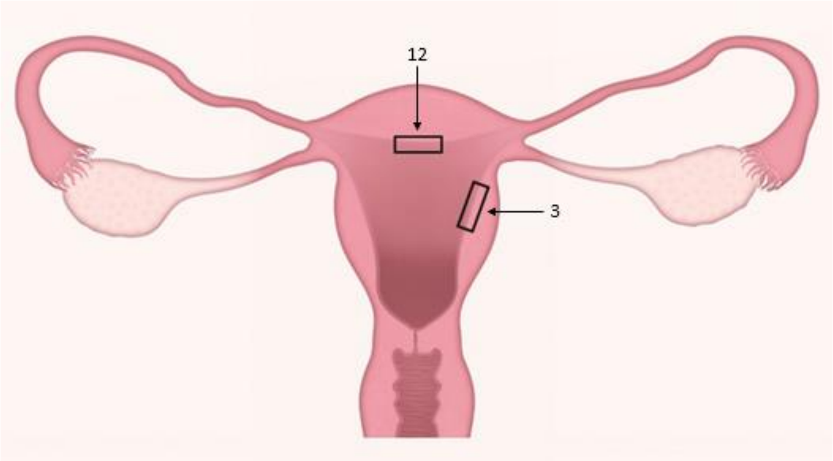

Figure S1: Endometrial sampling points.

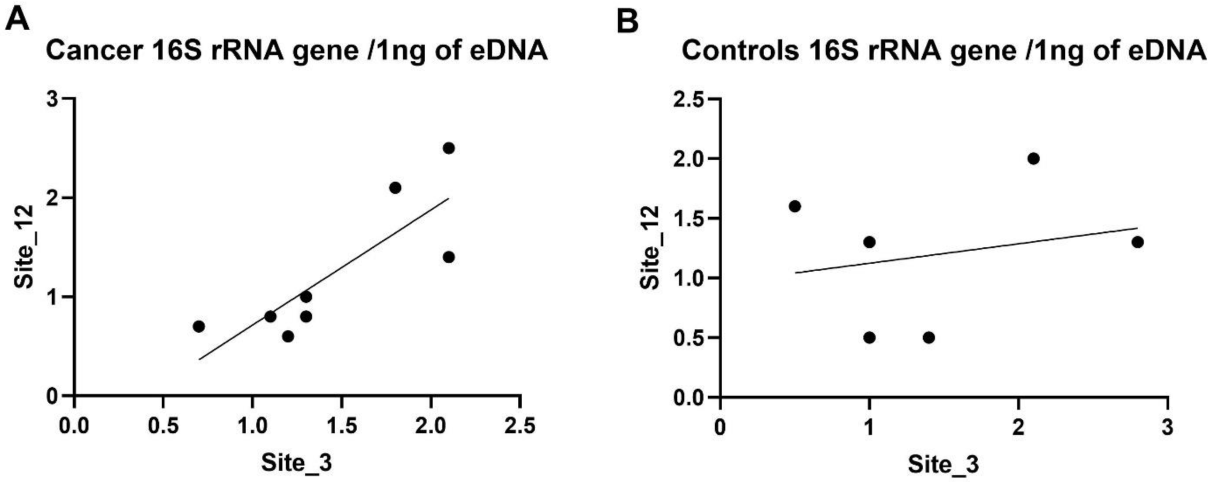

r	0.8241
95% confidence interval	0.2848 to 0.9671
R squared	0.6791
P value	
P (one-tailed)	0.0059

r	0.2293
95% confidence interval	-0.7154 to 0.8776
R squared	0.05260
P value	
P (one-tailed)	0.3310

Figure S2: Correlation analysis for absolute quantification of the 16S rRNA gene presence in two endometrial sites. **A**) Absolute quantification of the 16S rRNA gene correlates in two distinct endometrial sites (3 and 12) in cancer cases (Pearson r = 0.824, *p* = 0.0059. **B)** Absolute quantification of the 16S rRNA gene in two distinct endometrial sites (3 and 12) in control cases. The quantification does not correlate between the two sites (Pearson r = 0.2293, *p* = 0.331).

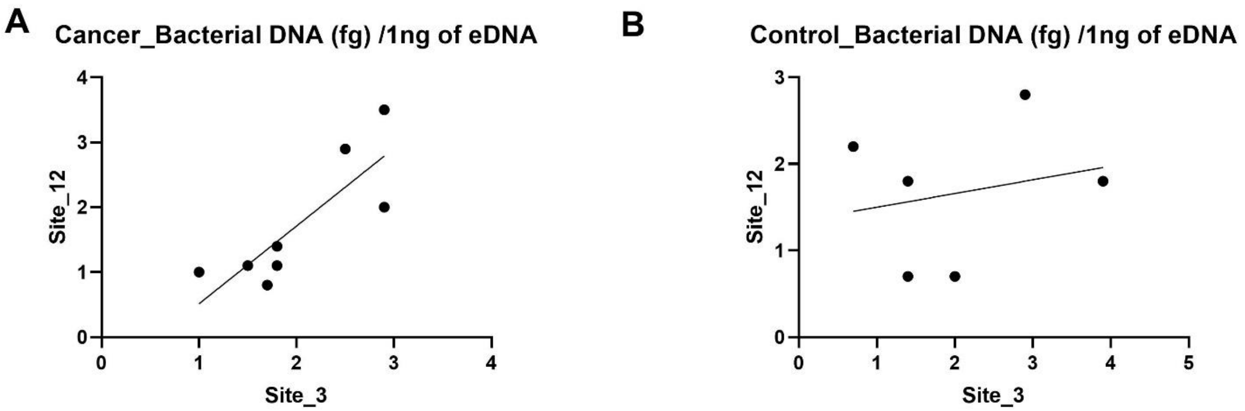

Pearson r	
r	0.8281
95% confidence interval	0.2963 to 0.9679
R squared	0.6857
P value	
P (one-tailed)	0.0056

Pearson r	
r	0.2220
95% confidence interval	-0.7191 to 0.8758
R squared	0.04928
P value	
P (one-tailed)	0.3362

Figure S3: Correlation analysis for absolute quantification of bacterial DNA in 1 ng of total eDNA in two endometrial sites**. A**) Absolute quantification of bacterial DNA correlates in two distinct endometrial sites (3 and 12) in cancer cases (Pearson r = 0.828, p = 0.0056. **B)** Absolute quantification of bacterial DNA in two distinct endometrial sites (3 and 12) in control cases. The quantification does not correlate between the two sites (Pearson r = 0.222, p = 0.336).

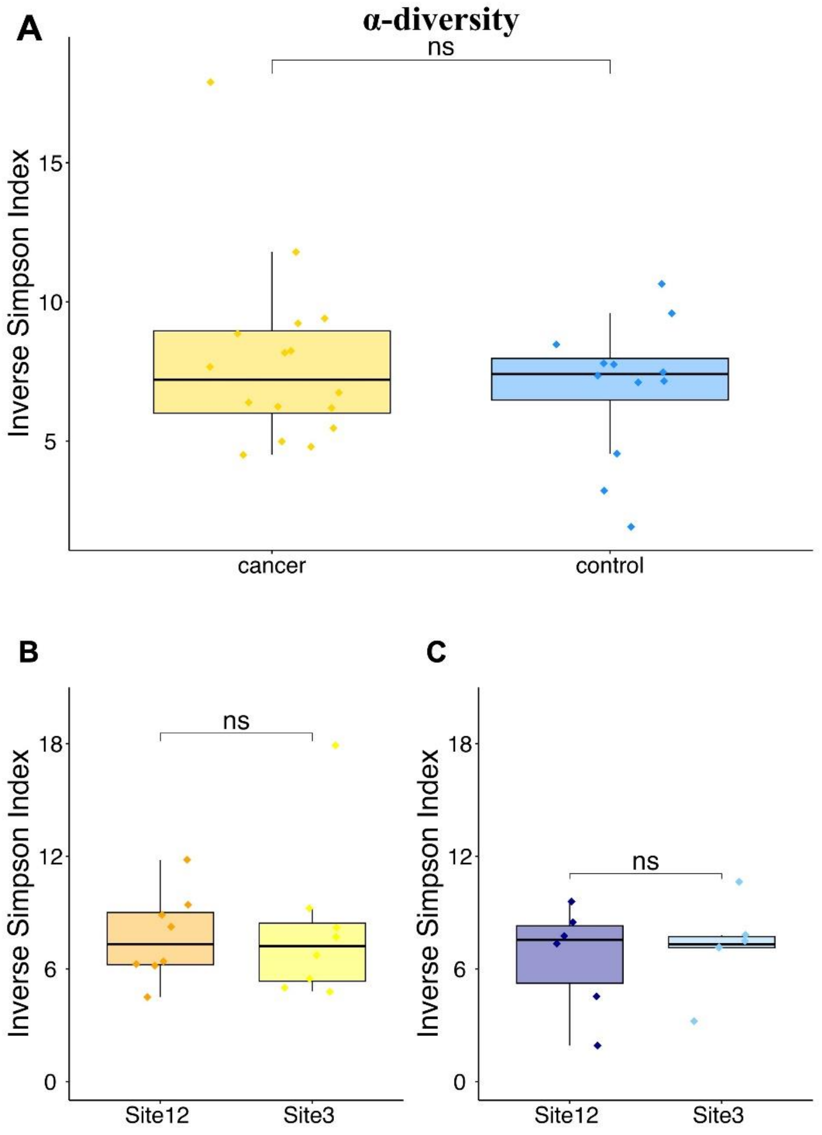

Figure S4: Shannon Simpson Index α-diversity values. A: Shannon Simpson Index for control and cancer cases; B) Shannon Simpson Index for 12 and 3 site in cancer cases; C) Shannon Simpson Index for 12 and 3 site in control cases.

Cancer	2K-3	2K-12	4K-3	4K-12	9K-3	9K-12	10K-3	10K-12	12K-3	12K-12	17K-3	17K-12	19K-3	19K-12	21K-3	21K-12	Average K3	Average K12
Ralstonia	18%	22%	14%	23%	16%	29%	14%	10%	15%	16%	17%	12%	13%	22%	39%	36%	18%	21%
Cutibacterium	24%	26%	45%	29%	44%	20%	55%	59%	13%	44%	52%	32%	49%	21%	24%	14%	38%	31%
Corynebacterium	2%	2%	6%	2%	6%	2%	8%	3%	20%	5%	10%	5%	6%	3%	6%	2%	8%	3%
Unclassified	6%	8%	9%	13%	5%	13%	3%	7%	3%	6%	3%	16%	1%	32%	3%	9%	4%	13%
Burkholderia-Caballeronia-Paraburkholderia	2%	1%	1%	2%	2%	2%	1%	2%	1%	2%	1%	2%		2%	2%	3%	1%	2%
Staphylococcus	4%	3%	2%		2%	1%	4%	5%	4%	2%	3%	4%	1%	2%	2%	2%	3%	3%
Streptococcus	3%	2%	3%	1%	1%		3%		2%	1%	2%	2%	1%		1%	1%	2%	1%
Pseudomonas		1%		1%		1%			13%	7%		1%		1%		2%	13%	2%
Sphingomonas	4%	4%			1%	1%		1%	1%		1%	2%	1%	1%		4%	1%	2%
Acinetobacter		2%	8%	9%								1%				2%	8%	3%
Escherichia-Shigella	21%	14%		3%	3%	20%		1%		1%	1%	4%		2%			8%	6%
Lactobacillus		1%		4%		2%		2%		1%				2%		1%	0%	2%

Control	8C-3	11C-3	14C-3	15C-3	16C-3	18C-3	8C-12	11C-12	14C-12	15C-12	16C-12	18C-12	Average C3	Average C12
Cutibacterium	48%	31%	35%	14%	36%	39%	6%	39%	13%	15%	31%	24%	34%	21%
Ralstonia	10%	12%	12%	54%	19%	13%	71%	10%	27%	43%	19%	26%	20%	33%
Corynebacterium	8%	16%	25%	7%	6%	23%	1%	3%	1%	1%	2%	2%	14%	2%
Lactobacillus	7%	1%					1%	5%	1%	7%	3%	7%	4%	4%
Unclassified	5%	5%	3%	4%	5%	3%	5%	18%	11%	14%	13%	12%	4%	12%
Staphylococcus	3%	3%	1%	4%	3%	2%	1%	4%	1%	1%	3%	1%	3%	2%
Streptococcus	1%	2%	3%			1%		1%	1%		2%	4%	2%	2%
Burkholderia-Caballeronia-Paraburkholderia	1%	3%		6%	1%	2%	5%	1%	3%	4%	2%	2%	3%	3%
Acinetobacter							1%			1%	1%		0%	1%
Escherichia-Shigella							3%	1%	2%	2%	10%	1%	0%	3%
Pseudomonas	1%	13%	5%	1%				5%	16%	1%	1%		5%	6%
Sphingomonas					1%	1%					2%		1%	2%
Gardnerella			1%						10%				1%	10%

Table S1: Bacterial genera with a percentage of presence ≥ 1% for all samples. Average of the percentage of presence of the bacterial genera present in all samples for both 3 and 12 site.

Control				Cancer			
**Genus**	**ef_lda**	**pvalue**	**padj**	**Genus**	**ef_lda**	**pvalue**	**padj**
g__0319-6G20	3,286985289	0,045315175	0,045315175	g__Sphingomonas	3,91436909	0,001153591	0,00115359
g__Alistipes	2,590561564	0,041640809	0,041640809	g__Cloacibacterium	3,34797541	0,029027308	0,02902731
g__cvE6	2,447276021	0,038031668	0,038031668	g__Peptoniphilus	3,34383966	0,028347339	0,02834734
g__Butyricicoccus	2,399692183	0,038031668	0,038031668	g__Haloquadratum	3,27556668	0,044865977	0,04486598
g__Phreatobacter	2,333095448	0,038031668	0,038031668				
g__Lachnospira	2,275540999	0,038031668	0,038031668				
g__Hydrotalea	2,229875485	0,014669886	0,014669886				

Table S2: LefSe analysis at genus level for control and cancer cases.
